# Application of Allogeneic Bone Marrow Cells in View of Residual Alloreactivity: Sirolimus but Not Cyclosporine Evolves Tolerogenic Properties

**DOI:** 10.1371/journal.pone.0119950

**Published:** 2015-04-02

**Authors:** Kai Timrott, Florian W. R. Vondran, Hueseyin Bektas, Jürgen Klempnauer, Mark D. Jäger

**Affiliations:** Klinik für Allgemein-, Viszeral- und Transplantationschirurgie, Medizinische Hochschule, Hannover, Germany; Beth Israel Deaconess Medical Center, Harvard Medical School, UNITED STATES

## Abstract

**Background:**

Application of bone marrow cells (BMC) is a promising strategy for tolerance induction, but usually requires strong depletion of the host immune system. This study evaluates the ability of immunosuppressants to evolve tolerogenic properties of BMC in view of residual alloreactivity.

**Methods:**

The rat model used a major histocompatibility complex (MHC) class II disparate bone marrow transplantation (BMT) setting (LEW.1AR1 (RT1^auu^) → LEW.1AR2 (RT1^aau^)). Heart grafts (LEW.1WR1 (RT1^uua^)) were disparate for the complete MHC to recipients and for MHC class I to BMC donors. Limited conditioning was performed by total body irradiation of 6 Gy. Cyclosporine (CsA) or Sirolimus (Srl) were administered for 14 or 28 days. Transplantation of heart grafts (HTx) was performed at day 16 or at day 100 after BMT. Chimerism and changes in the T cell pool were detected by flow cytometry.

**Results:**

Mixed chimeras accepted HTx indefinitely, although the composition of the regenerated T cell pool was not changed to a basically donor MHC class II haplotype. Non-chimeric animals rejected HTx spontaneously. BMC recipients, who received HTx during T cell recovery at day 16, accepted HTx only after pre-treatment with Srl, although chimerism was lost. CsA pre-treatment led to accelerated HTx rejection as did isolated application of BMC.

**Conclusion:**

Srl evolves tolerogenic properties of allogeneic BMC to achieve indefinite acceptance of partly MHC disparate HTx despite residual alloreactivity and in particular loss of chimerism.

## Introduction

Mixed hematopoietic chimerism after successful allogeneic bone marrow transplantation (BMT) is associated with donor-specific tolerance for solid organ grafts as shown in experimental studies and recently in first clinical trials [1–10]. Central clonal deletion of newly emerging T cell clones directed against donor antigens is generally accepted as the underlying mechanism. A prerequisite for this type of tolerance is eradication of peripheral mature T cell clones directed against donor antigens. This is usually achieved by rigid T cell depletion within the host. But near complete depletion of the T cell pool can evoke permanent immunological disadvantages or even malignant formation within the lymphopoeisis [11, 12]. From this point of view a rigid depletion of the host T cell pool should be avoided.

Moreover, despite intensive T cell depletion, residual immunity represents a critical barrier to tolerance. This barrier is even aggravated by heterologous immunity caused by T cell clones with cross-reactive specificities between pathogens and allogeneic antigens [13]. In some experimental studies, which investigated chimerism based tolerance, evidence for immunoregulation of residual alloreactivity or peripheral deletion has been reported [14–17]. To develop future strategies of tolerance induction based on application of allogeneic BMC, the interference between standard immunosuppressants, allogeneic BMC and residual host alloreactivity should be explored.

In the experimental model, the residual alloreactivity towards organ grafts will be increased, if bone marrow (BM) and subsequently transplanted solid organs are only matched in parts for their alloantigens. From previous studies using near to total T cell depletive conditioning protocols, the predominant role of MHC class II matching between BMC and solid organ grafts for tolerance development was identified [18–20]. Thus, the chosen rat model used limited matching between BM and heart grafts for MHC class II antigens only, but preserved MHC class I disparity between heart graft and BMC donors as well as complete MHC disparity between heart graft donors and recipients. Moreover, residual alloreactivity was preserved by incomplete depletion of the T cell pool using only 6 Gy total body irradiation (TBI).

Initially, CsA and Srl were compared for their properties to generate hematopoietic chimerism after MHC class II disparate BMT in view of residual alloreactivity. A special focus was laid on intrathymic engraftment of MHC class II expressing cells and resulting changes in the composition of the peripheral T cell pool. In recipients with a regenerated T cell pool, the survival of heart grafts differing additionally in MHC class I antigens from BMC was evaluated in dependence of chimerism and composition of the peripheral T cell pool. Finally, heart grafts were transplanted into BMT recipients two days after finishing a 14 days course of drug coverage to detect any immunosuppression dependent tolerogenic property of BMC despite chimerism loss.

## Methods

### Animals

Male inbred rats weighing 200 to 300 gram were purchased from and maintained in the central animal facility of Hannover Medical School, Hannover, Germany. We used the following rat strain combinations on LEW background: LEW.1AR1 rats (RT1A^a^, RT1B/D^u^, RT1C/E^u^) were used as BMC donors. LEW.1AR2 rats (RT1A^a^, RT1B/D^a^, RT1C/E^u^) served as recipients. In a control BMT setting, LEW.wt rats (RT1^l^ RT7^a^) served as recipients and LEW.7B rats (RT1^l^ RT7^b^, generously provided by K. Wonigeit, Hannover) as donors. LEW.1WR1 (RT1A^u^, RT1B/D^u^, RT1C/E^a^) and BN rats (RT1^n^) were used as heart donors. An overview of used strains and the corresponding MHC immunogenetics is given in **Table 1**. Detailed information are described elsewhere [21]. All animal procedures are approved by Niedersächsisches Landesamt für Verbraucherschutz und Lebensmittelsicherheit (animal protection number 02/528). Keeping of animals was provided in a specific pathogen free facility in a circadian rhythm of light and dark cycle with free access to water and food. For anaesthetics we applied ketamine and xylocain adopted to the actual body weight of every animal. To minimize suffering of the animals after the operation, metamizole was added to the water with free access. Animals were visited daily and palpation of the heart transplants was performed daily in the first four weeks after heart transplantation, in the further course three times per week. At the endpoint of the follow-up of heart transplantation, at the latest 200 days after, the animals were sacrificed by carbon dioxide inhalation and subsequent neck fracture. Respectively, this euthanizing procedure was used for all animals that acquired a poor clinical state (amongst others defined by loss of activity, increased sleepiness, and reduced food intake) or to the defined end points of the experiments (e.g. loss of graft function).

**Table 1:** MHC (RT1) immunogenetics of strain combinations used for heart transplantation to BMT recipients.

**strain combinations**		**MHC gene region**		
		**RT1A** *class I*	RT1B/D*class II*	RT1C/E*class I*
recipient	LEW.1AR2	a	a	u
BM donor	LEW.1AR1	a	u	u
heart donor	LEW.1WR1	u	u	a

Heart graft donor strain (LEW.1WR1) shares the u haplotype in the RT1B/D region with BMC donor strain (LEW.1AR1). It is disparate to BMC donor strain in the MHC class I encoding regions RT1A and RT1C/E. To the recipient strain (LEW1AR2) it is disparate in all MHC antigen complex encoding regions.

### Total body irradiation

Healthy rats were gamma-irradiated with a single dose of 6 Gy TBI with a linear accelerator (Philips MU15F/225 kV, Hamburg, Germany) one day before BMT. In the following chapters the day of TBI is defined as day -1 and the day of BMT and beginning of the application of immunosuppression is defined as day 0.

### Bone marrow transplantation

BMC were harvested from long-bones of euthanized LEW.1AR1 donor rats. Mature α/β TCR^+^ cells were removed *in vitro* from BMC inocula using mouse anti-α/β TCR mAb (R73, mouse IgG_1_, generously provided by K. Wonigeit, Hannover) and immunomagnetic beads (Dynabeads M-450, goat anti-mouse IgG, Dynal, Great Neck, NY). 1 x 10^8^ nucleated BMC were intravenously injected per recipient one day after TBI.

### Drug treatment

CsA (Sandimmun, Novartis Pharma AG) was mixed with olive oil to a final concentration of 15 mg/ml. Srl (Rapamune, Wyeth Pharmaceuticals) was used in a concentration of 1 mg/ml. CsA inhibits calcineurin and is responsible for activating the transcription of interleukin-2. Srl is an inhibitor of the complex of the mammalian Target Of Rapamycin (mTOR). Drugs were orally administered to BMT recipients every second day at 15 mg/kg body weight CsA and 2 mg/kg body weight Srl. These dosages have been shown to evoke adequate immunosuppressive potential in previous experiments of solid organ transplantation [22, 23]. For the first application of immunosuppression to the time point of BMT the dosages were doubled.

### Heart transplantation

HTx was performed either on day 16 or day 100 after BMT. Heart grafts were explanted, immediately perfused *ex vivo* with heparinized NaCl 0.9% solution and stored in ice-water. Within one hour after perfusion heart grafts were intraabdominally transplanted by standard technique as previously reported [24]. Graft rejection was defined as complete cessation of heart beat.

### Cell Preparation

Thymi and spleens were gently minced on a 70 μm stainless steel sieve. The resulting cell suspensions as well as heparinized blood were rendered free of red blood cells by an ammonium chloride solution (155 mM NH 4 Cl, 10 mM K HCO_3,_ 0.1 mM sodium EDTA). Blood cell counts were conducted by hematology analyzers (Sysmex XE2100, Diamond Diagnostics, USA). Spleen cell numbers were counted in a Neubauer counting chamber.

### Flow cytometry

**Fig 1:**
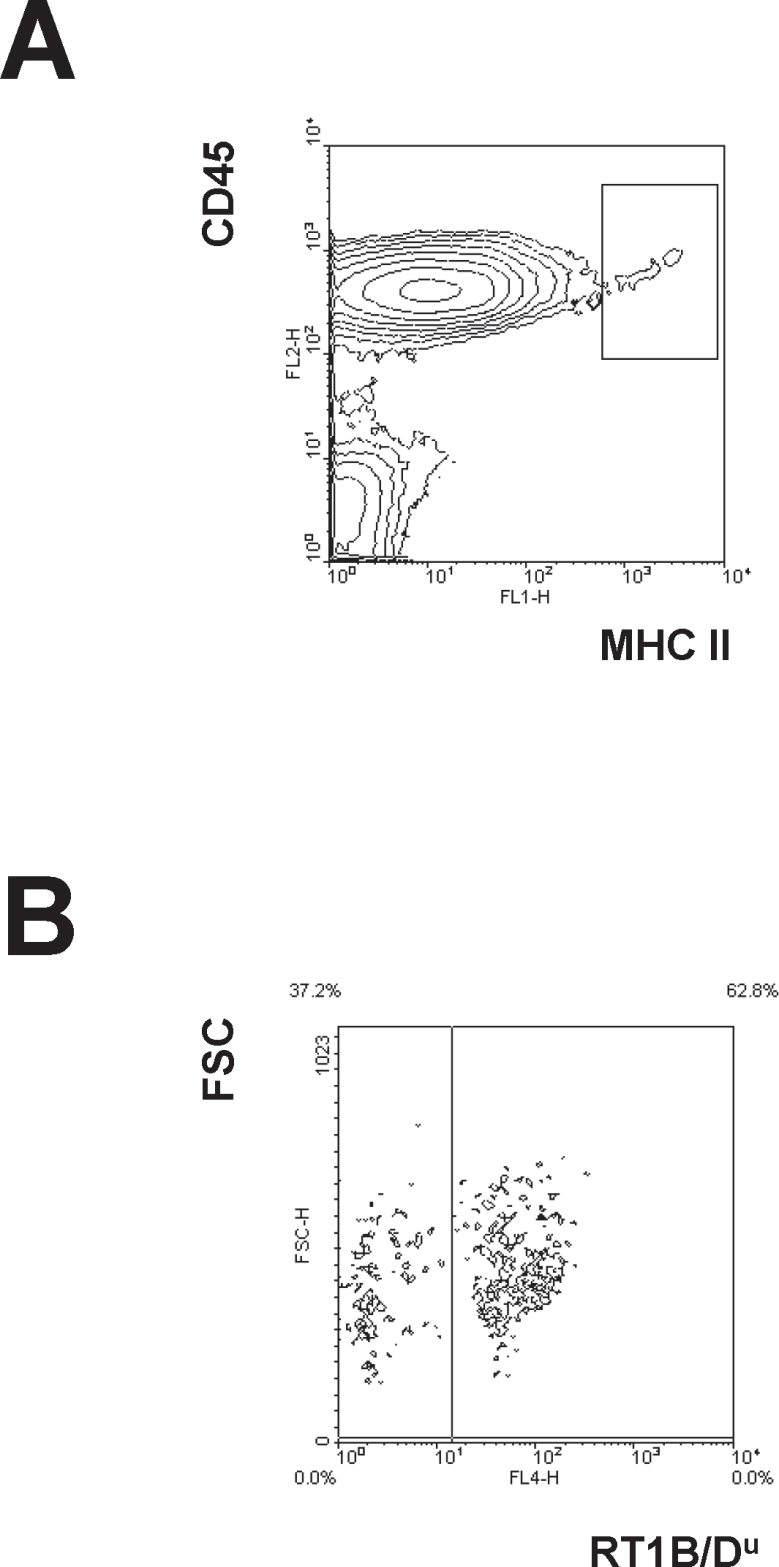
Detection of donor-derived MHC class II^high^ cells. Three colour flow cytometry was used to detect highly expressing MHC class II donor cells of BMT recipients. Mononuclear cells were pre-gated concerning scatter properties. (**A**) Leukocytes expressing high density of MHC class II antigens were gated using anti-CD45 mAb (OX-1) and anti-MHC class II mAb (OX-6). (**B**) Anti-RT1B/D^u^ mAb (1H1A) was used to detect cells of donor origin (1H1A^+^). Data from a representative chimeric recipient treated with 6 Gy and Srl for 28 days are shown.

For primary staining of blood leukocytes, spleen cells and thymocytes the following monoclonal antibodies were used: anti-α/β TCR mAb (R73, mouse IgG_1_), PE-conjugated anti-CD4 mAb (W3/25, mouse IgG_1_; both generously provided by K. Wonigeit, Hannover), PerCP-conjugated anti-CD8a mAb (OX-8, mouse IgG_1_, BD Pharmingen), anti-CD45 mAb (OX1, mouse IgG_1_, Pharmingen), anti-CD45RA mAb (OX-33, mouse IgG_1_, BD Pharmingen), anti CD103 mAb as marker for dendritic cells in rats (OX-62, mouse IgG_1_, BD Pharmingen), FITC-conjugated anti-MHC class II mAb (OX-6, mouse IgG_1_, BD Pharmingen) or biotin-conjugated anti-RT1B/D^u^ mAb (1H1A, rat IgG_1;_ generously provided by K. Wonigeit, Hannover). For detection of donor chimerism the u haplotype of donor-derived fractions (1H1A) from MHC class II expressing cells (OX-6) within CD45^+^ leukocytes (OX-1) was used (**Fig 1**).

Secondary stainings were performed with FITC-conjugated or PE-conjugated goat anti-mouse antibodies (BD Pharmingen). Blocking was performed with normal mouse serum. As fluorochromes streptavidin-PE or streptavidin-APC (BD Pharmingen) were used. Staining with Propidium iodide was used to exclude dead cells (PI, Sigma Chemical Co., St. Louis, MO).

All staining steps were performed on ice (4° Celsius). Flow cytometry was performed using a FACS Calibur (Becton Dickinson, Mountain View, CA).

### Statistics

Statistical analysis was performed using IBM SPSS Statistics 21.0 (Chicago, USA) applying the unpaired *t* test for the comparison of characteristics. The statistical significance was appointed at p<0.05. The data in the text and tables are presented as mean ± standard deviation or as otherwise stated.

## Results

### Persistence of α/β TCR^+^ cells and residual alloreactivity in irradiated rats

**Fig 2:**
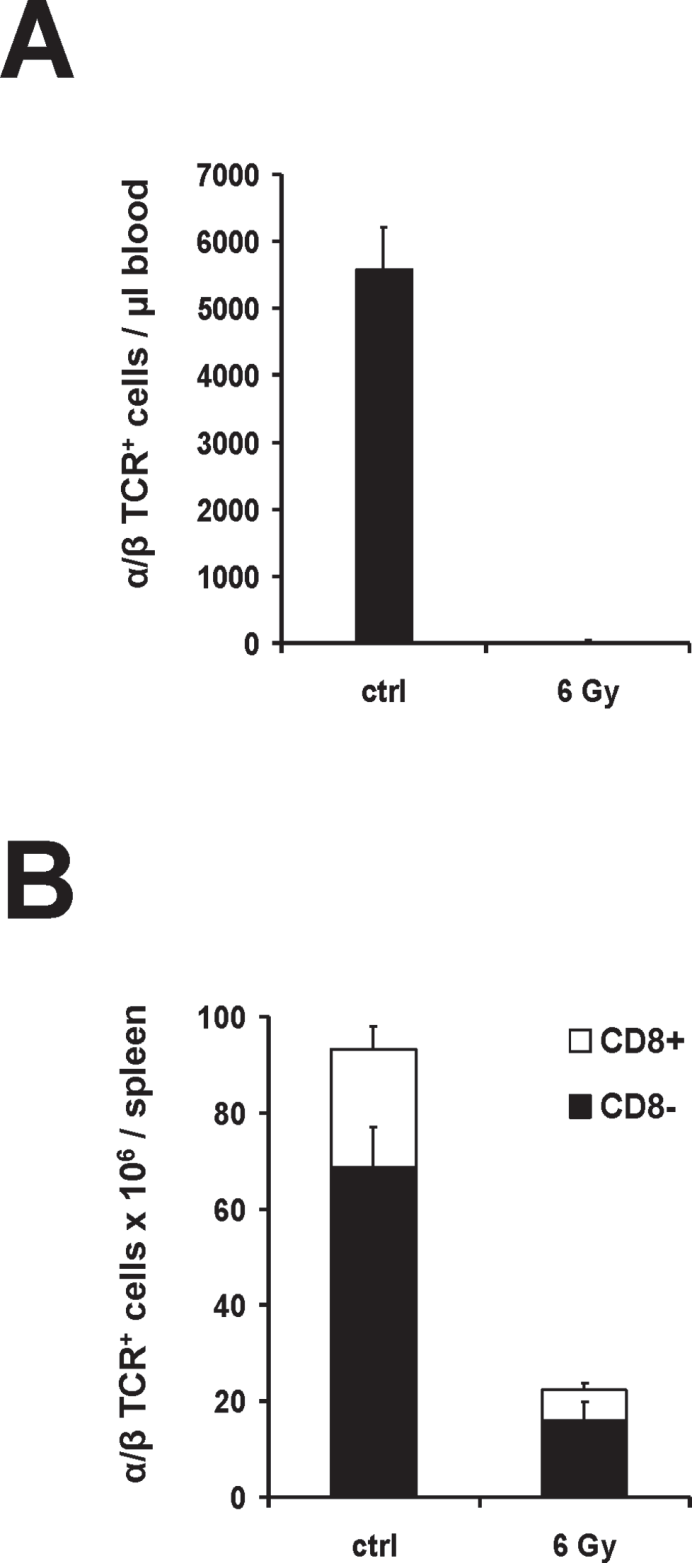
Effects of 6 Gy of TBI on T cell pool. Three male LEW.1AR2 rats were irradiated with 6 Gy of TBI. Analysis was performed on day 3 after TBI. Control animals of identical strain, gender and age did not receive any treatment (ctrl). After counting vital mononuclear cells per μl blood as well as per spleen, analyses were performed by flow cytometry. T cells with α/β TCR expression (R73^+^) were divided into CD8^+^ and CD8^-^. Propidium-iodid counter-staining was used to exclude avital cells. (**A**) Mean values of absolute numbers of α/β TCR+ cells in peripheral blood. (**B**) Absolute numbers of vital α/β TCR+ cells in spleen subdivided according to CD8 expression as mean values.

LEW.1AR2 rats were exposed to 6 Gy of TBI. At day 3, analysis of peripheral blood and spleens was performed. TBI reduced the fraction of α/β T cell receptor positive (TCR^+^) cells in blood to less than one percent of values in untreated control animals (**Fig 2A**). In spleens, absolute numbers of vital α/β TCR^+^ cells represented approximately 24 percent of values in untreated control animals. There were no significant differences for the proportion of CD8^+^ and CD8^-^ subsets (**Fig 2B**).

**Fig 3:**
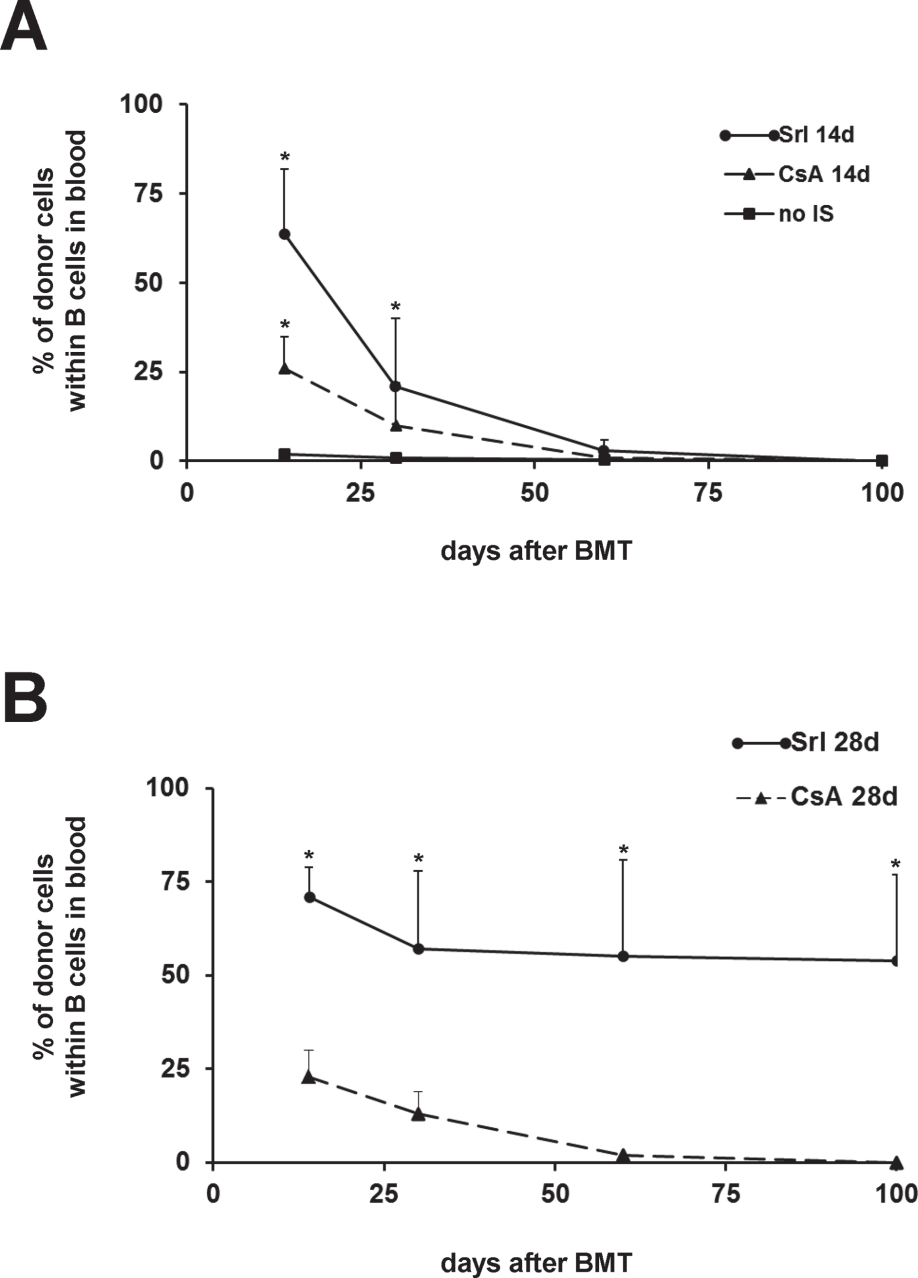
Courses of donor chimerism after MHC class II disparate BMT in recipients conditioned by 6 Gy of TBI and treated with immunosuppression. LEW.1AR2 male rats were irradiated with 6 Gy of TBI and received 1 x 10^8^ BMC from LEW.1AR1 donor rats one day after TBI. Recipients were additionally treated either with sirolimus (Srl) or cyclosporine A (CsA). Control animals did not receive any immunosuppression (no IS). Donor chimerism was detected within blood B cells by flow cytometry using donor-specific anti-RT1B/D^u^ mAb (1H1A) on days 14, 30, 60 and 100 post BMT. (**A**) Mean values of chimerism from groups receiving Srl for 14 days (Srl 14d; n = 13) or CsA for 14 days (CsA 14d; n = 11) as well as control animals (no IS; n = 11). (**B**) Mean values of chimerism from groups receiving Srl for 28 days (Srl 28d; n = 15) or CsA for 28 days (CsA 28d; n = 10). The statistical significance was appointed at * p < 0.05 for BMT groups receiving immunosuppression for 14 days and TBI alone (A) as well as for both groups receiving immunosuppression for 28 (B).

Irradiated rats received 1 x 10^8^ BMC intravenously from MHC class II disparate donors one day after TBI to assess residual alloreactivity. At day 14 no significant chimerism could be detected in peripheral blood (**Fig 3A and Table 2**). In contrast, MHC syngeneic BM grafts differing only in the CD45 antigen (LEW.7B rats (RT1^l^ RT7^b^) → LEW.wt rats (RT1^l^ RT7^a^)), induced stable high-grade chimerism as detected in blood at day 100 (68 +/- 7% donor leukocytes; n = 8; data not shown). Fully MHC disparate heart grafts were transplanted to irradiated, but not BMC transplanted recipients at day 16 after TBI. Five of six heart grafts were rejected at days 10, 15, 28 and twice at day 30 (**Table 2**). Thus, functional relevant residual alloreactivity was preserved after TBI of 6 Gy in the used transplant settings.

**Table 2:** Survival of heart grafts transplanted to irradiated BMT recipients at day 16 after BMT (during T cell recovery of recipients).

treatment group	% chimerism		heart graft survival (days)	MST	p value vs. 6 Gy
	day 14	day 100			
6 Gy			10, 15, 28, 30 x2, >100	28	
6 Gy BMT	2 +/- 1	0	11, 14 x2, 16	14	n.s.
6 Gy CsA			18, 21, 23 x2, 34	23	n.s.
6 Gy BMT CsA	32 +/- 8	0	14, 15, 23, 26, 29	23	n.s.*
6 Gy Srl			30, 35, 40 x2, >100	40	n.s.
6 Gy BMT Srl	62 +/- 22	0	> 100 x6	>100	< 0.005 ^#^

Setting of experiments and strain combinations are based on the Table 3. Groups are named according the pre-treatment by TBI, BMT and application of immunosuppression for 14 days starting at BMT. The degree of chimerism in blood of heart graft recipients is given for day 14 and day 100. Survival of each single heart graft transplanted at day 16 and mean survival times (MST) are given. The statistical significance was appointed at p < 0.05. P values of each group are compared to the 6 Gy TBI control group (n.s. = not significant). Additional statistical tests showed that * the immunosuppressive coverage by CsA did not prolong heart graft acceptance with BMT (6 Gy BMT CsA vs. 6 Gy BMT; p = 0.06) nor without BMT (6 Gy CsA vs. 6 Gy; p = 0.42). ^#^ Immunosuppressive coverage by Srl significantly prolonged heart graft acceptance after BMC application (6 Gy BMT Srl vs. 6 Gy BMT; p < 0.001), but not without BMC application (6 Gy Srl vs. 6 Gy; p = 0.48).

### Effect of Srl or CsA on generation of MHC class II disparate chimerism

Immunosuppressive coverage by Srl or CsA was added to prove that residual alloreactivity against MHC class II disparate BMC could be overcome. In the first set of experiments, drugs were administered from day 0 to 14. Although both immunosuppressants supported significant chimerism at days 14 and 30, no durable chimerism could be confirmed at later time points (**Fig 3A**).

By prolongation of immunosuppression until day 28 only Srl resulted in stable chimerism in 10 of 15 recipients (mean chimerism degree of 63 +/- 11% at day 100). In contrast, prolonged administration of CsA did not influence the course of chimerism (**Fig 3B**).

### Intrathymic chimerism and changes in the regenerated T cell pool

Thymi from representative BMT recipients were analysed for each group at day 100 after BMT. Fractions of donor-derived cells within leukocytes and within dendritic cells highly expressing MHC class II antigens were determined by flow cytometry using a donor MHC class II specific anti-RT1B/D^u^ mAb.

Naïve animals of LEW.1AR1 donor, LEW.1AR2 recipient as well as full chimeras generated by a lethal TBI dosage of 10 Gy were used as controls. These chimeras presented high-grade donor chimerism in the blood and in the thymus. In BMT recipients pre-treated with 6 Gy of TBI, only administration of Srl for 28 days led to a significant mixed chimerism in intrathymic MHC class II expressing hematopoietic cells (T**able 3**). In recipients receiving CsA for 28 days, intrathymic chimerism was just above the detection level of 0.5 percent given by flow cytometry. All other groups showed no detectible chimerism.

**Table 3:** Donor-derived fractions of MHC class II^high^ cells in the thymus of BMT recipients at day 100.

treatment group	1H1A^+^ cells (%)	
	CD45^+^ / MCH II^high^	CD103^+^ / MHC II^high^
6 Gy BMT	0.2 +/- 0.1	n.d.
6 Gy BMT CsA 14	0.2 +/- 0.1	n.d.
6 Gy BMT CsA 28	1.4 +/- 0.5	1.1 +/- 0.5
6 Gy BMT Srl 14	0.3 +/- 0.1	n.d.
6 Gy BMT Srl 28	49.0 +/- 8.1	42.7 +/- 15.3
10 Gy BMT	83.0 +/- 7.2	94.2 +/- 3.4

Three animals per group were analyzed by flow cytometry. Mean percentages of donor-derived fractions (1H1A^+^) from MHC class II highly expressing cells within CD45^+^ leukocytes and within dendritic cells (CD103^+^) are given. Reliable, high-grade chimerism in blood was present only in the 10 Gy BMT and the 6 Gy BMT Srl 28 groups. All other groups did not show any reliable chimerism (n.d. = not done).

**Fig 4:**
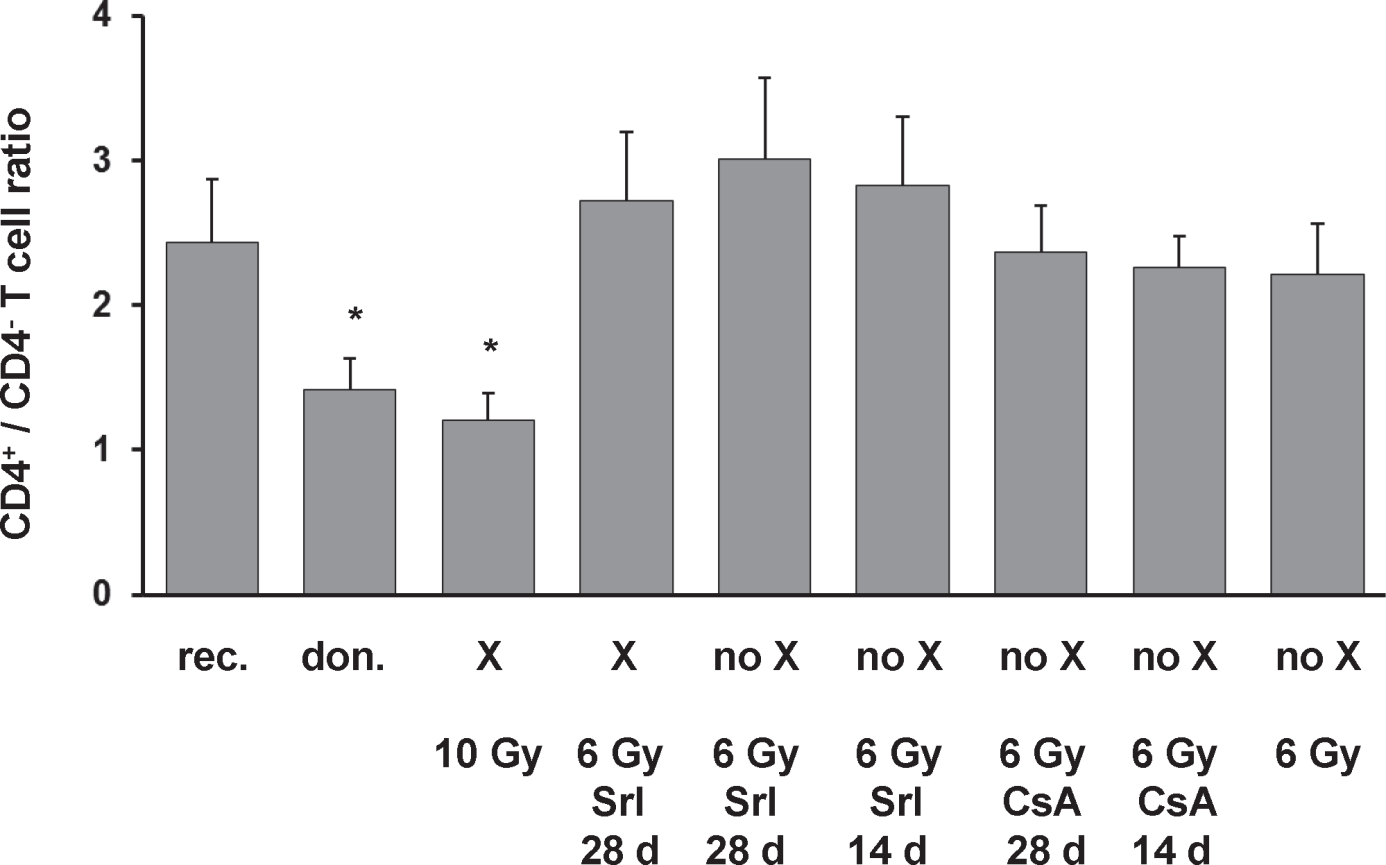
CD4^+^/CD4^-^ T cell ratio in peripheral blood of T cell regenerated BMT recipients at day 100. Peripheral blood of T cell regenerated BMT recipients at day 100 was analyzed by flow cytometry (n = 5 per group). Naïve rats of LEW.1AR1 donor and LEW.1AR2 recipient strain with same gender and similar age were analyzed as controls (n = 5 per group). Anti-α/β TCR mAb and anti-CD4 mAb were used. Mean values of CD4^+^/CD4^-^ ratios in α/β TCR^+^ cells are given. The type of chimerism is indicated (X = stable chimerism, no X = no persistent chimerism). Irradiation dosages and immunosuppressants (sirolimus: Srl; cyclosporine: CsA) including duration of application in days are given. The statistical significance was appointed at * p < 0.05 for naïve LEW.1AR1 as well as X 10 Gy group versus naïve LEW.1AR2 and all other groups.

In rat strains on LEW background, specific patterns of CD4^+^/CD4^-^ ratios within peripheral α/β TCR^+^ cells are associated with intrathymic expression of MHC class II a- and u-haplotypes [25] assuming that this ratio reflects thymocyte selection in dependence of the MHC class II haplotype. Thus, specific patterns of CD4^+^/CD4^-^ ratios within α/β TCR^+^ cells were demonstrated in naïve rats of BM donor strain LEW.1AR1 expressing exclusively the u-haplotype of MHC class II antigens and in naïve rats of BM recipient strain LEW.1AR2 expressing only the a-haplotype (**Fig 4**). At day 100 after BMT, analyzed recipients did not significantly differ in absolute numbers of α/β TCR^+^ cells in blood. In fully chimeric controls generated by lethal TBI dosage of 10 Gy the CD4^+^/CD4^-^ ratio corresponded to the high degree of donor-derived u-haplotype in intrathymic MHC class II expressing cells. In mixed chimeras generated by 6 Gy of TBI and Srl for 28 days, which contained similar intrathymic levels of a- and u-haplotype in MHC class II expressing cells, the CD4^+^/CD4^-^ ratio was similar to the a-haplotype specific pattern in naïve recipients and in all other recipient groups without significant intrathymic chimerism.

### Acceptance of fully MHC disparate heart grafts in BMT recipients with regenerated T cell pool

BMT recipients were tested for effects of BMC pre-treatment on acceptance of allogeneic heart grafts transplanted at day 100 after BMT. Heart graft donors differed in all MHC antigens from recipients and in MHC class I antigens from the BMC donors (**Table 1**). Thus, the BMC pre-treatment could not influence the alloreactivity against donor MHC class I antigens conserving a natural allobarrier between recipients and heart graft donors. This isolated MHC class I directed allobarrier caused chronic rejection in a control heart graft setting using naïve recipients (**Table 4**).

**Table 4:** Survival of heart grafts transplanted to irradiated BMT recipients at day 100 after BMT (after T cell regeneration of recipients).

treatment group	% chimerism		heart graft survival (days)	MST	p value vs. 6 Gy
	day 14	day 100			
naïve control (MHC I + II)			7, 8 x2, 9, 10	8	n.s.
naïve control (MHC I)			54, 73, 88, > 100 x2	88	
6 Gy			8, 10 x2, 12, 14	10	
6 Gy BMT	2 +/- 1	0	8, 9 x2, 10 x2	9	n.s.
6 Gy BMT CsA (14 days)	26 +/- 9	0	8, 11, 13, 14, 15	13	n.s.
6 Gy BMT CsA (28 days)	23 +/- 7	0	9, 13 x2, 17, 20	13	n.s.
6 Gy BMT Srl (14 days)	64 +/- 18	0	10, 12, 18, 19, 27, 32, 40	19	< 0.05 *
6 Gy BMT Srl (28 days)	71 +/- 8	63 +/- 11	> 100 x5	>100	< 0.001^#^
10 Gy BMT	84 +/- 9	98 +/- 2	> 100 x5	>100	< 0.001

The experimental groups are named according to the pre-treatment. Heart graft donors differed in all MHC antigens from recipients and in MHC class I antigens from the BMC donors. Control groups consist of naïve recipients receiving completely MHC disparate grafts (MHC I + II) or solely MHC class I disparate grafts (MHC I). Mean percentages of chimerism in blood are given at day 14 and day 100 after BMT. Survival of each single heart graft and mean survival times for each group (MST) are given. Statistical analyses were performed using two-sided, unpaired student´s t-test. Each group was compared with the 6 Gy control group (n.s. = not significant). Additional statistical tests showed that * transient chimeras treated with Srl (6 Gy BMT Srl (14 days)) accepted heart grafts significantly longer than transient chimeras treates with CsA (6 Gy BMT CsA (14 days)) (p = 0.047). ^#^ Prolonged Srl application resulted in significantly longer heart graft acceptance (6 Gy BMT Srl (28 days) vs. 6 Gy BMT Srl (14 days) (p < 0.001).

At day 100 after irradiation animals which received 6 Gy of TBI, but no BMC, regained alloreactivity to acutely reject MHC disparate heart grafts as rapid as untreated controls (**Table 4**). Full chimeras (10 Gy BMT), which switched their CD4^+^/CD4^-^ ratio within peripheral α/β TCR^+^ cells to a donor-specific pattern, and mixed chimeras (6 Gy BMT Srl 28 X), which retained a recipient-specific pattern of CD4^+^/CD4^-^ ratio, accepted MHC disparate heart grafts indefinitely. Both groups acutely rejected 3^rd^-party hearts of BN strain (RT1^n^; n = 3 each).

Non-chimeric BMT recipients, which received a transient immunosuppressive coverage with CsA or no immunosuppression, rejected their heart grafts acutely. In contrast, BMT recipients, which lost chimerism after immunosuppressive coverage with Srl for 14 days, showed significantly prolonged survival for heart grafts sharing the MHC class II antigens (MST: 19 days, p < 0.05), while acute rejection for 3^rd^ party control grafts of BN strain (MST: 10 days, n = 3) was observed. Differences in heart graft survival between non-chimeras covered with Srl and non-chimeras covered with CsA for 14 days were statistically significant (p < 0.05).

Comparing the application of Srl for 14 and 28 days after 6 Gy of TBI and BMT a significant prolongation of heart graft survival was observed for the application of Srl for 28 days (p < 0.001).

Thus, a beneficial effect evolving from Srl coverage of allogeneic BMC application to overcome residual alloreactivity could be assumed.

### Acceptance of fully MHC disparate heart grafts in BMT recipients during T cell recovery

To prove drug dependency of beneficial interference between allogeneic BMC and residual alloreactivity, secondary heart grafts were transplanted to BMT recipients two days after withdrawal of a 14 days lasting course of drug application. At this time point, the immune system had still to recover. Animals were regularly monitored for reconstitution of the T cell pool as well as donor chimerism in blood using flow cytometry (days 14, 30, 60 and 100).

Solely irradiated recipients retained sufficient residual alloreactivity to reject heart grafts in a delayed fashion (**Table 2**). Additional treatment with CsA did not prevent rejection of heart grafts, whereas additional treatment with Srl slightly prolonged survival times of subsequently transplanted heart grafts. Differences in these two treatment groups were not statistically significant (p = 0.122). In BMT recipients, who did not receive any additional immunosuppression, alloreactivity towards secondary heart grafts was enforced. CsA coverage of BMT did not influence the rejection of heart grafts. In contrast, Srl coverage of BMT induced indefinite acceptance of subsequently transplanted heart grafts despite loss of chimerism. The crucial interference of BMC and Srl, but not CsA was worked out by multiple statistical tests between experimental groups in **Table 2**. The group, which was treated with 6 Gy TBI, BMT and immunosuppressive coverage by Srl, kept alloreactivity against 3^rd^-party antigens like heart grafts from BN strain was confirmed by rejection at days 18, 21, 22 and 27 (data not shown).

These data underline that Srl evolves an immunomodulatory effect during the interference of allogeneic BMC and recovery of the host immune system to achieve acceptance of heart grafts despite residual alloreactivity.

## Discussion

In the present study, CsA and Srl were tested for their ability to induce hematopoietic chimerism and, moreover, to evolve tolerogenic effects of allogeneic BMC in the view of residual alloreactivity. The results show that Srl in contrast to CsA supports hematopoietic chimerism of MHC class II disparate BMC after partly T cell ablative conditioning and furthermore, evolves immunomodulatory properties of allogeneic BMC for indefinite acceptance of MHC disparate heart grafts.

The application of Srl or CsA following BMT enabled for considerable chimerism at day 14. However, durable chimerism was only achieved, when Srl was given for 28 days. A similar effect of Srl has been recently reported in an irradiation based BMT model using haploidentical mouse strains [6, 26]. Srl treated mice became stable chimeras, whereas CsA treated counterparts showed early loss of chimerism despite persistent immunosuppressive treatment. The authors speculated that CsA may inhibit TCR-induced tolerance induction within the periphery and the thymus. In contrast, Srl was promoted for prevention of graft rejection as well as for induction of tolerance and to support clonal deletion of anti-donor reactive T cells [27].

Indeed, successful clonal deletion of anti-donor reactive T cells plays an important role for stability of chimerism. The most essential prerequisite for clonal deletion of anti-donor reactive T cells is intrathymic engraftment of donor-derived MHC class II expressing cells [28]. In our study, we showed that only stable chimeras possessed a significant chimerism of intrathymic antigen presenting cells (APC) with high MHC class II expression at day 100. However, at day 14 Srl as well as CsA treated animals showed a marked and comparable intrathymic chimerism for APCs (data not shown). Thus, the most necessary prerequisite for successful intrathymic deletion of anti-donor MHC class II reactive T cell clones was theoretically given in both groups. However, only Srl, but not CsA could stabilize chimerism. These oppositional outcomes might be explainable by looking at the mechanisms of immunosuppressants and their influences on the thymocyte maturation process.

CsA blocks the action of calcineurin, thus, inhibiting TCR-mediated activation, which is required for correct intrathymic clonal deletion [29]. This could result in disturbed intrathymic deletion of thymocytes with high avidity to presented antigens. As a consequence, rejection of donor MHC class II expressing cells would occur. This could explain the loss of chimerism despite prolonged CsA treatment. In contrast, Srl does not interfere with the TCR-mediated activation, but inhibits T cell proliferation at the G_1_-S stage [30]. Thus, thymocyte maturation is blocked at an early stage before clonal deletion occurs [31]. Thereby, Srl provokes a certain thymic atrophy, but does not disturb processes of thymocyte selection [32]. At this time point, leakage of thymic filling and of intrathymic alloreactivity could further support thymic seeding of donor-derived APC. Thus, an optimized clonal deletion process would result after drug withdrawal.

The persistence of a considerable intrathymic chimerism in regenerated chimeras should result in durable clonal deletion of anti-donor MHC class II reactive T cells. The CD4^+^/CD4^-^ ratio within peripheral α/β TCR^+^ cells on LEW background has been shown to be dependent on the haplotype of intrathymically expressed MHC antigens and thus to differ between different strains [25]. Although analysis in naïve rats of donor as well as recipient strain confirmed a MHC class II haplotype specific pattern, the CD4^+^/CD4^-^ ratio changed to donor-type pattern in full chimeras generated by 10 Gy TBI, but not in mixed chimeras generated by 6 Gy TBI and Srl for 28 days. This observed recipient-type pattern in mixed chimeras could partly result from persistence of significant degree of recipient thymopoiesis in view of the non-lymphoablative conditioning which gives rise to recipient phenotype thymocytes / lymphocytes as well as from incomplete negative selection due to interaction with limited numbers of donor-derived APC corresponding to the fact that the intrathymic extent of donor MHC class II expression determines the efficacy of clonal deletion [33]. Moreover, in the peripheral lymphoid system more T cell clones will survive irradiation with 6 Gy than irradiation with 10 Gy [34]. These radiation resistant T cell clones will undergo proliferation driven by homeostasis as well as by antigen contact and might considerably contribute to the emerging T cell pool.

Taken together it seems likely that persistence of significant degree of recipient thymopoiesis incomplete intrathymic clonal deletion as well as persistence and proliferation of radiation resistant mature T cell clones in peripheral lymphoid tissues are responsible for the unchanged pattern of CD4^+^/CD4^-^ ratio within peripheral α/β TCR^+^ cells in mixed chimeras. In this context, it was especially remarkable that mixed chimeras accepted heart grafts indefinitely, which differ additionally in MHC class I alloantigens. Thus, the MHC class I allobarrier, which leads to chronic rejection in a heart transplant setting using naïve rats, was already neutralized in mixed chimeras. Since 3^rd^ party heart grafts were acutely rejected, any alloantigen specific immunomodulatory effect could be generated by coverage of allogeneic BMT with Srl. This hypothesis for any immunomodulatory properties of allogeneic BMC evolved by Srl was further strengthened by the fact that heart grafts presented significantly prolonged survival in T cell regenerated non-chimeric recipients only when Srl was administered for BMT coverage.

By transplanting heart grafts into BMT recipients during recovery of the T cell pool, but after withdrawal of the immunosuppression, the suggested supportive effect of Srl, but not CsA for evolvement of tolerogenic properties by allogeneic BMC was observed. In contrast to Srl, CsA coverage of BMC application did not prolong heart graft acceptance according to the reported inhibition of TCR-induced tolerance [35, 36]. This inefficacy of CsA could be explained by a disturbed thymic regeneration under CsA causing increased homeostasis-driven proliferation of memory T cell clones due to reduced suppression of extrathymic T cell regeneration pathways [37]. In contrast, treatment with Srl in the absence of BMT already prolonged heart graft survival. This might be due to a general proliferation blockade slowing down the regeneration of the T cell pool. For the thymus dependent pathway of T cell regeneration this has been already shown [31]. But also the thymus-independent homeostasis-driven pathway of T cell regeneration could be inhibited by the anti-proliferative effect of Srl leading to a delayed increase in memory T cell numbers. Moreover, Srl has been shown to increase the fraction of CD4^+^CD25^+^ regulatory T cells during T cell reconstitution after irradiation [38]. Concerning preceded antigen exposure by BMC a preserved dominance of alloantigen-driven expansion to regulatory T cells under the influence of Srl was shown by *in vitro* studies for human CD4^+^CD25^+^CD27^+^ regulatory T cells [39]. Moreover, apoptosis of peripheral alloreactive T cell clones could still occur with Srl as shown in studies investigating mechanisms of peripheral TCR-induced tolerance [36].

Although at this point the exact mechanism of immunomodulation by Srl was not clarified, the conclusion that Srl is beneficial to evolve tolerogenic properties of allogeneic BMC in view of residual alloreactivity can be drawn. Thus, Srl might qualify for tolerizing strategies using allogeneic BMC in the face of residual anti-donor alloreactivity.

## Supporting Information

S1 ARRIVE Checklist A brief description of what this file is. (PDF)
